# Fabrication and characterization of novel electrospun nanofibers based on grass pea (*Lathyrus sativus* L.) protein isolate loaded with sumac (*Rhus coriaria* L.) extract

**DOI:** 10.1016/j.crfs.2024.100891

**Published:** 2024-11-09

**Authors:** Marzieh Rezaei, Nasser Sedaghat, Sara Hedayati, Mohammad-Taghi Golmakani

**Affiliations:** a Department of Food Science and Technology, School of Agriculture, Ferdowsi University of Mashhad, Mashhad, Iran; b Nutrition Research Center, School of Nutrition and Food Sciences, Shiraz University of Medical Sciences, Shiraz, Iran; c Department of Food Science and Technology, School of Agriculture, Shiraz University, Shiraz, Iran

**Keywords:** Active nanofibers, Electrospinning, Grass pea protein isolate, Sumac extract

## Abstract

In this study, sumac extract was utilized as an active ingredient and combined with grass pea protein isolate and polyvinyl alcohol to produce novel active nanofiber mats using an electrospinning technique. First, nanofiber mats were fabricated by different ratios (100:0, 90:10, 70:30, 50:50, 30:70, 10:90, 0:100) of grass pea protein isolate and polyvinyl alcohol. The characterization of nanofiber mats revealed that the nanofibers with a polymer ratio of 50:50 had appropriate mechanical properties and presented a fibrous and uniform morphology. Therefore, the 50:50 polymer solution ratio was selected to produce active nanofibers by adding different amounts (0%, 2%, and 4% (w/v)) of sumac extract. The average diameters of nanofibers decreased from 150 ± 31 to 122 ± 25, and 105 ± 19 nm, by increasing the concentration of sumac extract. Based on the SEM results, the electrospun nanofibers exhibited a bead-free and smooth surface. The FTIR and XRD analyses indicated the presence of intermolecular hydrogen bonds between the components. The antioxidant activity of the nanofibers was confirmed by DPPH analysis and ranged between 3.33% and 68.75%. Additionally, the antimicrobial test results indicated that the nanofibers with the highest sumac concentration (4%) displayed inhibitory activity against *Staphylococcus aureus*, resulting in an inhibition zone of 10 mm. The optimal treatment of this study was grass pea protein isolate: polyvinyl alcohol ratio of 50:50 containing 4% sumac extract which can be used as a natural antimicrobial and antioxidant agent.

## Highlights

• Grass pea protein isolate (GPPI) nanofibers were fabricated by electrospinning.
• Polyvinyl alcohol (PVA) was blended with GPPI to improve its spinnability.
• Sumac extract (SE) was incorporated into nanofibers as an active agent.
• SE-loaded GPPI nanofibers showed antimicrobial and antioxidant activities.

## Graphical abstract

**Figure:**
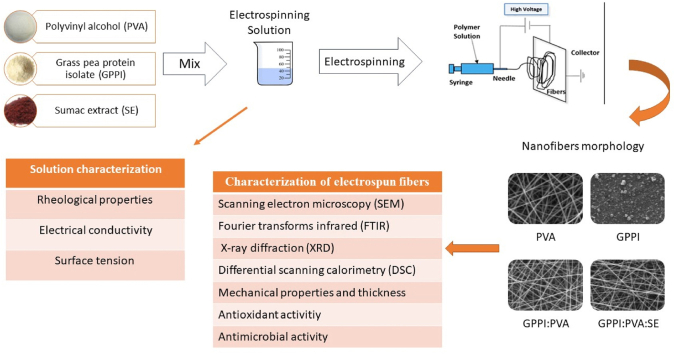

## 1 Introduction

Packaging plays an undeniable role in improving the quality, safety, and shelf life of food products. Electrospinning is frequently applied to produce nanofiber mats from polymers suitable for food applications (Aman Mohammadi et al., 2024). The worldwide rise in antimicrobial resistance, the restrictions of antibiotic use, and health issues regarding synthetic antimicrobial agents and chemical preservatives for controlling microbial growth in foods are important factors driving the interest in natural antimicrobials and preservatives that are both safe and effective (Beikzadeh et al., 2020). The inclusion of natural bioactive compounds in packaging materials can help shelf-life extension. However, it is difficult to use bioactive substances in food packaging because they can easily decompose and lose their functionality. Therefore, it is very important to develop effective strategies to protect bioactive compounds and control their release. The encapsulation of bioactive substances into electrospun packaging materials enhances their stability and effectiveness (Tarahi et al., 2024). However, choosing the right biopolymers and active compounds is a critical step in the production of nanofibers, which determines their applications. The interest in replacing synthetic packaging materials with those made from plant-based polymers is constantly increasing, primarily due to their superior sustainability and reduced environmental impact. Therefore, the potential of plant proteins as packaging materials is being explored in numerous studies (Hadidi et al., 2023).

Proteins are suitable materials for the production of films and coatings due to their exceptional characteristics, such as their abundance, high nutritional value, remarkable film-forming characteristics, and good barrier properties (Zhang et al., 2023). Plant proteins, particularly legume proteins, are being consumed more widely across the world due to their nutritional and functional properties (Tarahi et al., 2024). Grass pea (*Lathyrus sativus* L.) is a member of the Fabaceae family that is drought-resistant and can be grown in arid regions. As the demand for environmentally friendly protein sources increases, grass pea is increasingly recognized as a fantastic option for sustainable food production. Grass pea is a rich source of essential amino acids like leucine, lysine, arginine, glutamic acid, and aspartic acid. Grass pea seeds are known as a novel protein source, as they contain around 30% protein with a great amino acid profile. The grass pea protein isolate (GPPI) is made up of different types of proteins. The protein profile of GPPI is primarily composed of globulins (66%), glutelins (15%), albumin (14%), and prolamin (5%) (Feyzi et al., 2018; Ghobadi et al., 2021). Nevertheless, like many other protein sources, it is a challenging task to produce electrospun nanofibers using only GPPI. Blending GPPI with other polymers is a promising approach to make it spinnable and fabricate nanofibers with desirable features that can be used in drug delivery and food packaging (Huang et al., 2022). Polyvinyl alcohol (PVA) is a synthetic polymer with excellent spinnability. The PVA nanofibers have remarkable mechanical characteristics, biodegradability, biocompatibility, and gas barrier properties, and several studies have demonstrated that they can improve the spinning properties of biopolymers (Hosseini et al., 2019; Maftoonazad et al., 2019).

Anthocyanins are considered valuable components due to their enhanced health-promoting properties and biological activities. However, they are highly susceptible to temperature, light, solvent, metal ions, pH, and decomposition during the shelf-life period (Aman Mohammadi et al., 2023). Notably, the encapsulation of such compounds not only maintains their antioxidant and antimicrobial activities but also improves their thermal stability (Sharif et al., 2022). Sumac (*Rhus coriaria* L.) is a spice with numerous health benefits that can be used in both food supplements and medicines. It contains different bioactive compounds with antioxidant properties and antimicrobial activity against a wide range of microorganisms. Previous studies have detected anthocyanins, flavones, tannins, and organic acids in sumac (Alsamri et al., 2021). The combination of electrospinning technology and bioactive plant extracts can be used as an effective strategy to extend the shelf life of perishable products. This can be attributed to biodegradability, safety, sustained release of active compounds, better antimicrobial activity, and barrier properties (Sameen et al., 2022). Due to the unique properties of sumac and GPPI, the encapsulation of sumac extract (SE) as a rich source of anthocyanins into GPPI using electrospinning is a promising approach to enhance its stability and protect it from environmental factors, leading to the fabrication of bioactive nanofibers with great antioxidant and antimicrobial properties. To the best of our knowledge, there is no available data on the preparation of electrospun nanofibers using GPPI and SE. Therefore, the purpose of this study was to develop GPPI electrospun nanofibers incorporating SE and investigate their morphological, structural, thermal, mechanical, antibacterial, and antioxidant properties.

## 2 Materials and methods

### 2.1 Materials

The grass pea seeds and sumac fruits were purchased at a shop situated in Shiraz, Iran. Pure ethanol and methanol were obtained from Pars Alcohol Company (Eghlid, Iran). Glacial acetic acid, polyvinyl alcohol, NaOH, HCl, DPPH (2,2-diphenyl-1-picrylhydrazyl), and Mueller Hinton agar were gained from Sigma-Aldrich. All chemicals were employed without any additional purification and were of analytical grade.

### 2.2 Sumac extract preparation

This process included soaking 5 g of ground sumac fruit in 75 mL of aqueous ethanol 20% (v/v) for 5 hours with stirring at 40 ^°^C. Then the mixture was cooled and filtered through filter paper. The extract was concentrated with a rotary evaporator (Laborota 4001 efficient, Heidolph, Schwabach, Germany) at 45 ^°^C to remove the solvent, and the remaining fraction was freeze-dried (Kossah et al., 2011).

### 2.3 Preparation of grass pea protein isolates (GPPI)

An alkaline extraction/acid precipitation process was used to extract grass pea protein isolate. Grass pea seeds were ground by a laboratory grinder and passed through a sieve (mesh 60). The grass pea flour was mixed with distilled water in a ratio of 1:10, and 1 M NaOH was added to increase the pH of the slurry to 9.5 while magnetically stirring for 2 hours. Next, the undissolved matter was separated by centrifugation, and the pH of the supernatant was adjusted to 4.5 by adding 1 M HCl. After that, it was centrifuged at a speed of 10000×*g* for 10 min. The pellet was washed two times with distilled water and recentrifuged under the same conditions. After that, the pH of the pellet was increased to 7 by adding NaOH solution (1 M) and was freeze-dried (Feyzi et al., 2018).

### 2.4 Preparation of electrospinning solutions

PVA solution 7% (w/v) was prepared by dissolving 7 g PVA in 100 mL 50% (v/v) acetic acid solution and stirring at 85 ^°^C for 2 h. Also, GPPI solution 7% (w/v) was prepared by dissolving in a 50% (v/v) acetic acid solution and stirring for 1 h at 80 ^°^C. Then the spinning solutions were prepared by mixing different ratios (100:0, 90:10, 70:30, 50:50, 30:70, 10:90, 0:100) of GPPI:PVA solutions and stirring for 30 minutes. Based on the pre-tests, the GPPI:PVA with a ratio of 50:50 showed the best spinning properties, thus being selected for adding SE.

### 2.5 Solution characterization

#### 2.5.1 Electrical conductivity (EC) and surface tension (ST)

The electrical conductivity of spinning solutions was determined using an electrical conductivity meter (CONSORT C933, Turnhout, Belgium) at 25 ^°^C. Placing two electrodes with equal voltage in the solution led to the production of current; the resulting current is a direct reflection of the solution's conductive properties (Souri et al., 2023). The surface tension was measured using a force tensiometer (Krüss K100 Tensiometer, Hamburg, Germany) before the electrospinning process (Yao et al., 2022).

#### 2.5.2 Rheological properties of spinning solutions

The rheological properties of the solutions were determined through a rheometer (Rheometer mcr 302 Anton Paar, Austria). The analysis of the rheological properties was conducted by examining the steady shear behavior at a shear rate ranging from 0 to 100 s^−1^ under a temperature of 25 ^°^C. Additionally, the dynamic rheological behavior was studied under the following conditions: shear rate of 0.1%, frequency range of 0.1–100 Hz, at 25 ^°^C. Subsequently, the elastic modulus (G′) and viscous modulus (G″) were determined.

### 2.6 Electrospinning process

The electrospinning process was performed using an electrospinning machine (Side-by-side Electrospinning Unit, dual pump, ES2000, Tehran, Iran). A 2.5-mL plastic syringe with a 23-gauge metal needle was used for electrospinning of biopolymeric solution under the following conditions (flow rate: 0.5 mL/h, voltage: 20 kV, and distance between needle and collector = 15 cm, at room temperature and 50% relative humidity). The drum collector was covered with aluminum foil to facilitate the isolation of electrospun fibers for further analysis.

### 2.7 Characterization of electrospun fibers

#### 2.7.1 Scanning electron microscopy (SEM)

Scanning electron microscope (TESCAN-Vega 3, Brno, Czech Republic) determined fiber morphology. Fibers were placed on the horizontal part and fixed with conductive glue. To increase the conductivity, the samples were coated with gold before testing (Yao et al., 2022). ImageJ (Version 5.3.5, Ostend, Belgium) software was applied to determine the average diameter of fibers with a random selection of at least 90 nanofibers (Golmakani et al., 2024).

#### 2.7.2 Fourier transforms infrared (FTIR)

A FTIR spectrometer (Tensor II, Bruker, Germany) in the range of 4000 to 400 cm^−1^ wavelength and a resolution of 4 cm^−1^ was applied to characterize the structural interactions between polymers and the samples were subjected to 32 scans (Yao et al., 2022).

#### 2.7.3 X-ray diffraction (XRD)

The X-ray diffraction tests were conducted with an X-ray diffractometer (D8 Advance Bruker, Germany) using CuK radiation. The tests were performed within the scanning angle 2ϴ range of 10–70^°^, with a voltage of 40 mV and a current of 40 mA.

#### 2.7.4 Differential scanning calorimetry (DSC)

The DSC (DSC-131 evo, Setaram, France) was used to examine the thermal properties of the fibers in an enclosed environment filled with nitrogen gas flowing at a rate of 30 mL/min. The samples underwent a gradual temperature increase from 25 to 400 ^°^C, with a heating rate of 10 ^°^C/min (Sharif et al., 2019).

#### 2.7.5 Mechanical properties and thickness

A texture analyzer (TA-XT2, Stable Microsystems, Surry, UK) was used to measure the mechanical properties of the samples, including tensile strength (TS) with Eq. (1):

Eq. (1) TS = F/L.X.

where TS (MPa), F (N), L (mm), and X (mm) are the tensile strength, axial tensile force, width, and thickness of the fiber mats, respectively.

and elongation at break (EB) was calculated using Eq. (2):

Eq. (2) E (%) = (L_1_-L_0_)/L_0_ × 100

where E (%), L_0_ (mm), and L_1_ (mm) are the elongation-at-break, the initial length of the sample, and the length of the sample when it breaks, respectively.

The thickness of nanofiber mats was measured five times by a micrometer (Mitutoyo No. 293–766, Tokyo, Japan), and the average values were calculated (Yao et al., 2022).

#### 2.7.6 Antioxidant activities of fibers

To assess the antioxidant activity of the nanofibers, a 2,2-diphenyl-1-picrylhydrazyl (DPPH) radical scavenging assay was performed. Various concentrations (1.25, 2.5, 5, 10, 20, and 40 μg/mL) of solutions containing 0.1 mM DPPH, 70% methanol, and SE (∼4 mg) or nanofibers (∼20 mg) were utilized. The UV–Vis spectrophotometer was utilized to measure the absorbance of the solutions at 517 nm after 30 min. The antioxidant activity was calculated using Eq. (3).

Eq. (3) Antioxidant activity (%) = [(A _control_ – A _sample_)/A _control_] × 100

where A _sample_ and A _control_ indicate the absorbance of DPPH solution in the presence and absence of SE, respectively.

#### 2.7.7 Antimicrobial activity

The disc diffusion method was used to evaluate the antimicrobial activity of nanofiber. The microorganisms utilized in the experiment included *Escherichia coli* (*E. coli*) and *Staphylococcus aureus* (*S. aureus*) bacteria. Before performing the test, the nanofiber mats with a diameter of 10 mm underwent sterilization using an ultraviolet lamp (Hajjari et al., 2023).

### 2.8 Statistical analysis

The data analysis was done using IBM SPSS Statistics 27 (SPSS Inc., Chicago, IL) with analysis of variance (ANOVA) and Duncan's multiple range test (*P* values < 0.05).

## 3 Results and discussion

### 3.1 Solution characteristics

The spinnability of biopolymer solutions is strongly influenced by solution properties such as viscosity, surface tension, and electrical conductivity. These properties are key factors in determining the ideal conditions for obtaining continuous fibers.

#### 3.1.1 Rheological behavior

Rheological tests are used to explain how materials deform when subjected to external forces. By studying rheological behavior, we can gain insights into the structural interaction between the components in the spinning solution, thereby providing a deeper understanding of the physical properties of nanofibers (Liang et al., 2018). To achieve successful polymer electrospinning and the formation of a Taylor cone, it is necessary to have an adequate level of viscosity. However, excessive viscosity hinders the spinning of polymer solutions.

**Fig. 1:**
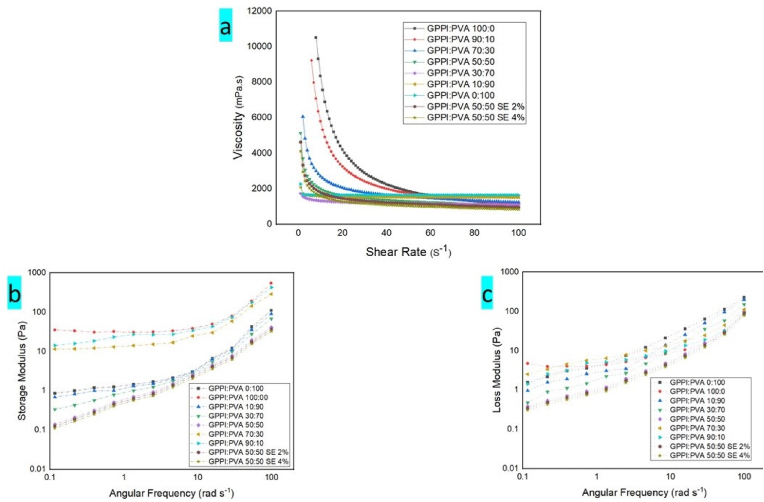
Rheological properties of spinning solutions: (a) Apparent viscosity/shear rate, (b) Storage modulus (G′), and (c) Loss modulus (G″). PVA: Polyvinyl alcohol, GPPI: grass pea protein isolate, and SE: sumac extract.

The rheological properties of the solutions are illustrated in Fig. 1. It can be observed that all solutions, except for PVA, demonstrated shear thinning characteristics, suggesting that they were pseudoplastic fluids (El Miri et al., 2015). This behavior is probably due to the breakdown of the entanglements in the biopolymeric solution network. Another possible reason is the changes in the structure of polymeric complexes caused by shear forces (Azarikia and Abbasi, 2016). The rise in shear forces disturbs the interactions within the biopolymer network and results in the formation of particle clusters or droplet aggregates that decrease the apparent viscosity (Long et al., 2012). As the PVA content in GPPI:PVA solutions increased, the apparent viscosity exhibited a decreasing trend. Nevertheless, the pseudoplasticity was reduced, and at high shear rates, the viscosity of samples with a higher ratio of PVA was greater (Fig. 1a). The resistance to shear forces is very critical when a biopolymer is subjected to high deformation rates in the electrospinning because it is an indication of its spinnability. SE incorporation into the GPPI:PVA solution resulted in lower viscosities. The decrease in viscosity with the addition of SE can be due to two reasons: first, the reduction of the amount of polymer per unit volume, and second, the weakening of biopolymer chain linkages with the addition of SE. Other studies have reported similar findings after the addition of anthocyanin to biopolymeric solutions (Zeng et al., 2023; Liu et al., 2023).

It is essential to assess the dynamic rheological properties to provide insights into the structural features and interactions among the solution's components. The solutions were evaluated for their dynamic rheological characteristics using storage modulus (G′) and loss modulus (G″) measurements by frequency sweep test (Fig. 1b and c). In all of the solutions, there were progressive rises in G′ and G″ as the frequency increased. The GPPI solution showed a G′ value greater than the G″ value, indicating that it was more elastic. While, the G″ value of PVA solution was greater than its G′ value. Consequently, the G′ modulus was decreased by increasing the PVA ratio in the composite solutions. The results of this study are consistent with the findings of Zhang and Pan (2020) about silk fibroin/polyvinyl alcohol blended solutions. Upon the integration of SE, the G′ and G″ moduli were decreased. These changes are in agreement with the shear steady measurement results.

#### 3.1.2 Electrical conductivity

The EC of polymeric solutions is an important parameter in the development of smooth and bead-free electrospun fibers. Low EC of the solution produces larger-diameter fibers, while high EC of the spinning solutions leads to excessive stretching, which can produce very fine fibers or prevent a uniform spinning process. Therefore, the EC of the spinning solution must be high enough to increase the electrostatic repulsion, facilitate the jetting of the spinning solution, and produce sufficient elongation and homogenous electrospun fibers (Seethu et al., 2020).

Based on the obtained results, the EC of the pure PVA solution was approximately 580.40 ± 3.45 (μS/cm), and the electrical conductivity of the mixed solutions showed a significant decline (*P* < 0.05) with increasing the PVA content (Table 1). The same pattern was identified in the case of sunflower protein isolate/PVA (Shanesazzadeh et al., 2018), and pea protein isolate/pullulan (Aguilar-Vázquez et al., 2018) nanofibers. By incorporating SE, a gradual increase in EC was observed. This result may be connected to the higher amounts of ionizable groups in the spinning solutions. Similar results were reported by Cruz et al. (2024), when red onion bulb extract was added to zein nanofibers.

**Table 1:** The effect of different ratios of GPPI:PVA and SE on the electrical conductivity and surface tension of spinning solutions.

Polymeric solution	Conductivity (μS/cm)	Surface tension (mN/m)
GPPI:PVA 0:100	580.40 ± 3.45 ^g^	46.22 ± 4.31^g^
GPPI:PVA 100:0	1283.33 ± 7.63 ^a^	91.39 ± 5.06 ^a^
GPPI:PVA 10:90	638.50 ± 10.73 ^f^	49.58 ± 4.03 ^fg^
GPPI:PVA 30:70	777.56 ± 16.34 ^e^	54.38 ± 5.99 ^ef^
GPPI:PVA 50:50	881.26 ± 12.90 ^d^	59.45 ± 3.61 ^de^
GPPI:PVA 70:30	969.86 ± 16.70 ^c^	73.05 ± 2.22 ^c^
GPPI:PVA 90:10	1176.66 ± 7.63 ^b^	83.25 ± 2.67 ^b^
GPPI:PVA 50:50 SE 2%	886.33 ± 12.13 ^d^	60.27 ± 1.34 ^de^
GPPI:PVA 50:50 SE 4%	901.18 ± 1.71^d^	63.16 ± 2.11^d^

PVA: Polyvinyl alcohol, GPPI: grass pea protein isolate, and SE: sumac extract. Different letters on the same column exhibited significant differences (*P* < 0.05).

#### 3.1.3 Surface tension

The surface tension of a solution is extremely important in electrospinning; when the internal electrostatic repulsion between molecules overcomes their surface tension, electrospinning begins. To ensure the formation of stable jets during electrospinning, it is important to measure surface tension. Generally, the reduction of surface tension increases the interactions between polymer and solvent molecules and results in the formation of smooth fibers. However, a lower surface tension of the spinning solution does not necessarily guarantee the success of the electrospinning process. Therefore, surface tension should be evaluated alongside other key parameters (Zaitoon and Lim, 2020). The data in Table 1 demonstrate the surface tension values for various GPPI:PVA ratios. GPPI:PVA with a ratio of 0:100 exhibited the lowest surface tension value (46.22 mN/m), and GPPI:PVA with a ratio of 100:0 showed the highest value (91.39 mN/m). For solutions with GPPI:PVA mixtures, increasing the amount of GPPI caused a remarkable rise in the surface tension. The addition of SE slightly increased the surface tension of the solutions. The maximum amount of SE that can be used for electrospinning is about 4%. Similar results have been reported for solutions of bean protein polyvinyl alcohol (El Halal et al., 2019) sunflower seed protein polyvinyl alcohol (Shanesazzadeh et al., 2018) and starch-based nanofiber mats with roselle anthocyanins (Lv et al., 2024).

### 3.2 SEM

SEM is utilized to evaluate the surface morphology, analyze the distribution of fiber diameter, determine the optimal operating parameters, and determine the determine the composition of the polymer solution for nanofiber production (Dey et al., 2020). The SEM was utilized to study the morphological characteristics of nanofibers. The SEM images revealed that GPPI cannot form fibers and only micro/nano-sized particles were produced during the electrospinning of GPPI solution. Despite the high electrical conductivity of the GPPI solution, it could not have the capability to form fibers. This is likely due to the complex network composition of grass pea protein and also its globular conformation,d PVA were combined in a ratio of 70:30, bead-free and uniform fibers were produced. which lacks sufficient chain entanglement for fiber formation (El Halal et al., 2019). Correspondingly, previous studies have demonstrated that it is impossible to produce fibers from pure sunflower and bean proteins (Shanesazzadeh et al., 2018; El Halal et al., 2019). PVA has been employed in numerous studies to facilitate the production of fibers (Shanesazzadeh et al., 2018; Gutschmidt et al., 2021). The electrospinning of the GPPI:PVA solution with a ratio of 90:10 resulted in non-uniform and beaded nanofibers. When GPPI and PVA were combined in a ratio of 70:30, bead-free and uniform fibers were produced. As the GPPI ratio decreased, the nanofibers transformed shape from beaded structures to uniform fibers, and their average diameter increased.

**Fig. 2:**
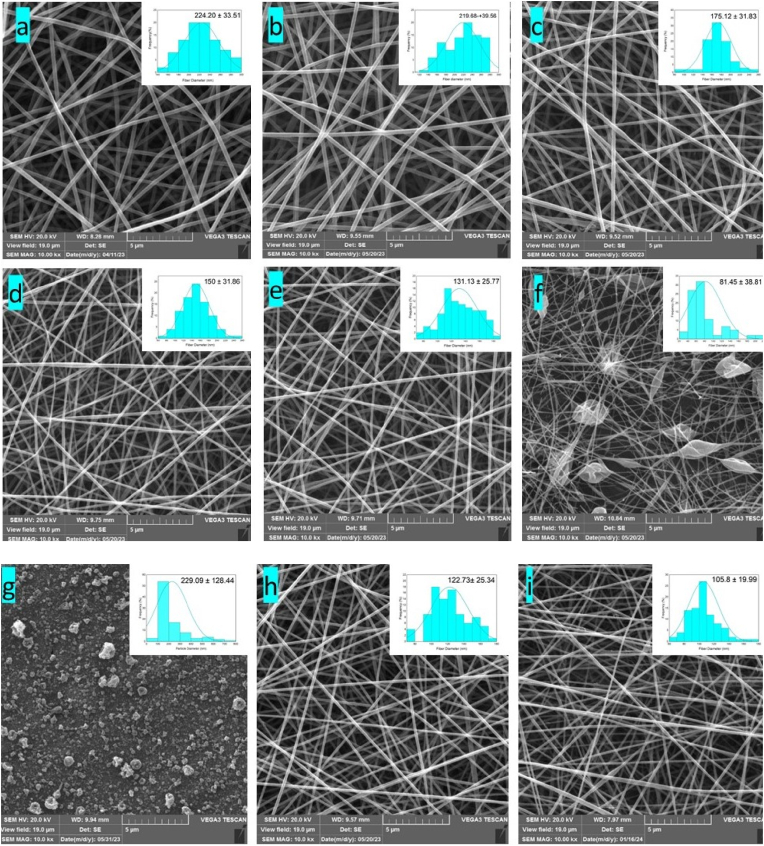
SEM image of (a) PVA, (b) GPPI:PVA 10:90, (c) GPPI:PVA 30:70, (d) GPPI:PVA 50:50, (e) GPPI:PVA 70:30, (f) GPPI:PVA 90:10, (g) GPPI, (h) GPPI:PVA 50:50 SE 2%, and (i) GPPI:PVA 50:50 SE 4%, PVA: Polyvinyl alcohol, GPPI: grass pea protein isolate, and SE: sumac extract.

The mean fiber diameter and the diameter distribution of electrospun fibers are presented in Fig. 2. The average diameter of PVA, GPPI:PVA (10:90, 30:70, 50:50, 70:30) fibers were 224.2, 219.68, 175.12, 150, and 131.13 nm, respectively. The increase of fiber diameter by increasing the PVA ratio may be due to the increase of solution viscosity and reduction of solution conductivity. These factors decrease with stretching of the polymer jet during electrospinning, resulting in larger fibers. Similarly, Gutschmidt et al. (2021), reported that the diameter of electrospun fibers with higher ratios of soy protein is lower than those with lower protein ratios. Based on our pretreatments, the GPPI:PVA ratio of 50:50 was selected as the optimal treatment for adding SE. As indicated in Fig. 2 h, and i, the incorporation of SE had substantial effects on the morphology of nanofibers, and the fiber diameter was decreased by increasing the concentration of SE. According to the findings of Cruz et al. (2024), polymer solutions with reduced viscosity contribute to a reduction in molecular entanglement, which in turn leads to a decrease in the fiber diameter. Our study revealed a similar trend, indicating that a decrease in the viscosity of the polymeric solutions led to smaller fiber diameter. Moreover, an increase in SE concentration was associated with enhanced electrical conductivity in the polymeric solutions, subsequently causing a reduction in the diameter of the nanofibers.

The diameter of the GPPI:PVA nanofibers with a ratio of 50:50 and SE of 2% was 122.73 nm and reached 105.8 nm by the incorporation of 4% SE. This decrease can be attributed to the increased weight of the polymer jet ejected from the capillary, which led to higher elongation. Also, the incorporation of SE increased the electrical conductivity and electrostatic field traction, which makes the Taylor cone form more quickly and the fibers stretch more easily (He et al., 2022). These results were consistent with the findings of Cruz et al. (2024), who stated that the diameter of the fibers decreased with the increase in the amount of red onion bulb extract in the zein fibers.

### 3.3 FTIR analysis

**Fig. 3:**
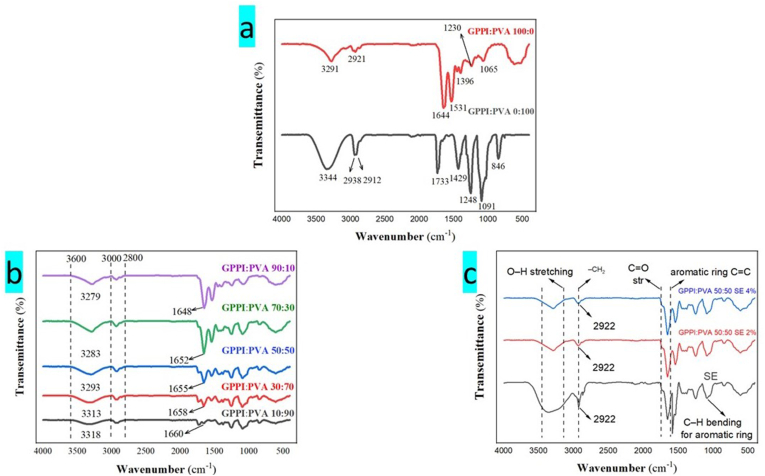
FTIR spectra of (a) PVA and GPPI, (b) nanofibers with different ratios of GPPI:PVA, and (c) SE and GPPI:PVA nanofibers with different concentrations of SE, PVA: Polyvinyl alcohol, GPPI: grass pea protein isolate, and SE: sumac extract.

FTIR analysis was conducted on the nanofibers and their components to assess their possible interactions. The FTIR spectra of PVA (Fig. 3a), showed a peak at 3344 cm^−1^ that can be attributed to O-H stretching vibrations, while the observed bands at 2938 and 2912 cm^−1^ can be attributed to the vibrations of C-H stretching. The peaks observed at 1248 and 1091 cm^−1^ are assigned to the stretching vibrations of carbon-hydrogen (C-H) and carbon-oxygen (C-O) bonds (Allafchian et al., 2022). Furthermore, the peaks detected at 1733, 1429, 1248, and 1091 cm^−1^ were associated with the stretching of C-O bonds in vinyl acetate groups that were not subjected to hydrolysis, as well as the asymmetric stretching of CH_2_ and symmetric bending, vibrations of C-O-C, and asymmetric stretching of C-O bonds. Additionally, the peak at 846 cm^−1^ corresponded to the stretching of C-C bonds (Maftoonazad et al., 2019; Gutschmidt et al., 2021). The FTIR spectrum of pure GPPI exhibited a distinctive peak at 3291 cm^−1^, indicating the presence of free and bonded O-H and N-H groups (Fig. 3a). These groups can form hydrogen bonds with the carbonyl group of the peptide bond in the protein. The 2921 cm^−1^ shoulder corresponds to the stretching vibrations of CH and NH_2_, while the absorption at 1644 cm^−1^ is attributed to the stretching vibrations of the amide I carbonyl group (C=O).

The distinctive peak at 1531 cm^−1^ indicates the bending movement of N-H groups and the stretching motion of C-N groups in amide II. The presence of a band at 1396 and 1230 cm^−1^ signifies the movement or vibration of the amide III region. This movement can be attributed to either the C-N and N-H groups of the attached amide or the CH_2_ group of glycine. The C-O stretching vibration is said to be the cause of the peak at 1065 cm^−1^ (Ghobadi et al., 2020). The interaction between PVA and GPPI led to a marked reduction in the intensity of the O-H peaks, transitioning from 3600 cm^−1^–3000 cm^−1^. This alteration indicates the potential formation of extensive intermolecular hydrogen bonds between GPPI and PVA (Maftoonazad et al., 2019). In the hybrid nanofibers, the amide II peaks moved to higher wave numbers, from 1531 cm^−1^ in GPPI to 1648-1660 cm^−1^ in the hybrid nanofibers (Fig. 3b). All of the hybrid GPPI:PVA nanofiber ratios (10:90, 30:70, 50:50, 70:30, and 90:10) presented functional groups associated with PVA and GPPI. After the addition of GPPI, the C=O stretching peak of PVA shifted from 1733 cm^−1^ to a range of 1660-1648 cm^−1^. This alteration is likely due to hydrogen bonding interactions between the hydroxyl groups in PVA and the amide groups in GPPI (Gutschmidt et al., 2021).

This suggests that GPPI and PVA can mix and interact with each other (Shanesazzadeh et al., 2018). As shown in Fig. 3b, as the GPPI content increased, the absorption peaks attributed to the stretching vibrations of OH bonds in hybrid nanofibers experienced a shift towards lower wave numbers (3318–3279 cm^−1^). Similarly, Dehnad et al. (2023), and Gutschmidt et al. (2021), indicated that the incorporation of soy protein isolate into PVA nanofiber resulted in a shift of the stretching vibrations of OH bonds to lower wave numbers. This indicates the formation of intermolecular hydrogen bonds between the protein and PVA. In GPPI:PVA:SE nanofibers with varying amounts of SE (2% and 4% (w/v)) had special bands at 1000-950 cm^−1^ because of hydrogen bonds between GPPI:PVA and SE (Fig. 3c). This shows that the GPPI:PVA and SE are interacting well, and the SE is being encapsulated effectively. A band narrowing, centered around 3300 cm^−1^, was detected in these spectra that can be ascribed to the elongation of the O-H and N-H bonds compared to GPPI. This narrowing might be a result of intermolecular interactions, such as hydrogen bonding, between the polymer and anthocyanins in SE. The band located at 2922 cm1 is representative of the C-H stretching associated with aliphatic groups. The band observed at approximately 1635 cm^−1^ was associated with the C-C stretching of the phenyl ring, which was present in substantial amounts as polyphenolic components in SE (Cruz et al., 2024). The absence of a peak gap in the hybrid nanofibers indicates that GPPI, PVA, and SE were consistently distributed throughout the fibers. The results of this research align with the conclusions drawn by Tavassoli et al. (2023), who stated that the incorporation of sumac anthocyanins into pectin film increased the peak associated with O-H bonds. This increase may result from the formation of hydrogen bonds between the hydroxyl groups of sumac anthocyanins and pectin.

### 3.4 XRD

**Fig. 4:**
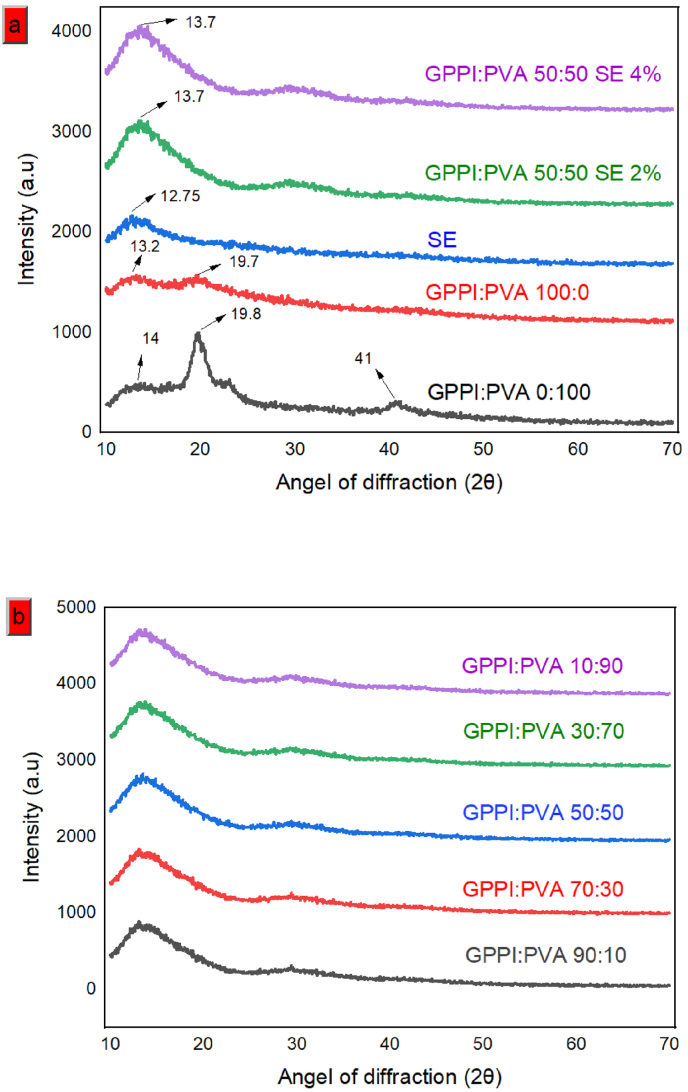
XRD patterns of: (a) PVA, GPPI, SE, GPPI:PVA nanofibers with different concentrations of SE, and (b) nanofibers with ratios of GPPI:PVA. PVA: Polyvinyl alcohol, GPPI: grass pea protein isolate, and SE: sumac extract.

The objective of XRD analysis is to identify whether biopolymers possess amorphous or crystalline characteristics. Crystallinity serves as a potential indicator of mechanical strength in electrospun fibers since materials with a crystalline structure typically exhibit higher strength in comparison to those with an amorphous structure (Zhang et al., 2020). The XRD patterns of fibers can be visualized in Fig. 4. The diffraction pattern of PVA exhibits peaks at approximately 14^°^ and 19.8^°^. Furthermore, a diffraction peak was detected at 41^°^, signifying that PVA exhibits a partially crystalline structure (Fig. 4a). This crystallinity is attributable to the robust intermolecular interactions within the PVA polymer network (Zhai et al., 2017). Similarly, Dey et al. (2020), reported that there are several sharp peaks in XRD patterns of PVA, indicating its crystalline nature. The GPPI crystalline pattern included two prominent peaks at angles of 13.2^°^ and 19.7^°^, these peaks represent the α-helix and β-sheet structures (Ghobadi et al., 2020). Chen et al. (2013), also reported the presence of distinct diffraction peaks at an angle of 2θ of 10^°^ and 22^°^ in soybean protein and stated that these peaks reflect 7S (α-helix) and 11S (β-sheet) globulins within the chain structure of polypeptides. The XRD spectrum of GPPI:PVA shows a characteristic peak at 13^°^; this suggests the presence of the composite fibers having a partially crystalline structure. After adding SE, the XRD pattern of the fibers did not show any new peaks or changes in the recorded peaks. It indicated that SE was evenly distributed within the fiber matrix with hydrogen bonding and did not change the crystalline structure of nanofibers (Lv et al., 2024). Similar patterns were reported by Pakseresht et al. (2023), and Hu et al. (2024), for active Cerish fructan-sumac extract composite films and blueberry anthocyanin-based wheat gluten protein films, respectively.

### 3.5 DSC

**Fig. 5:**
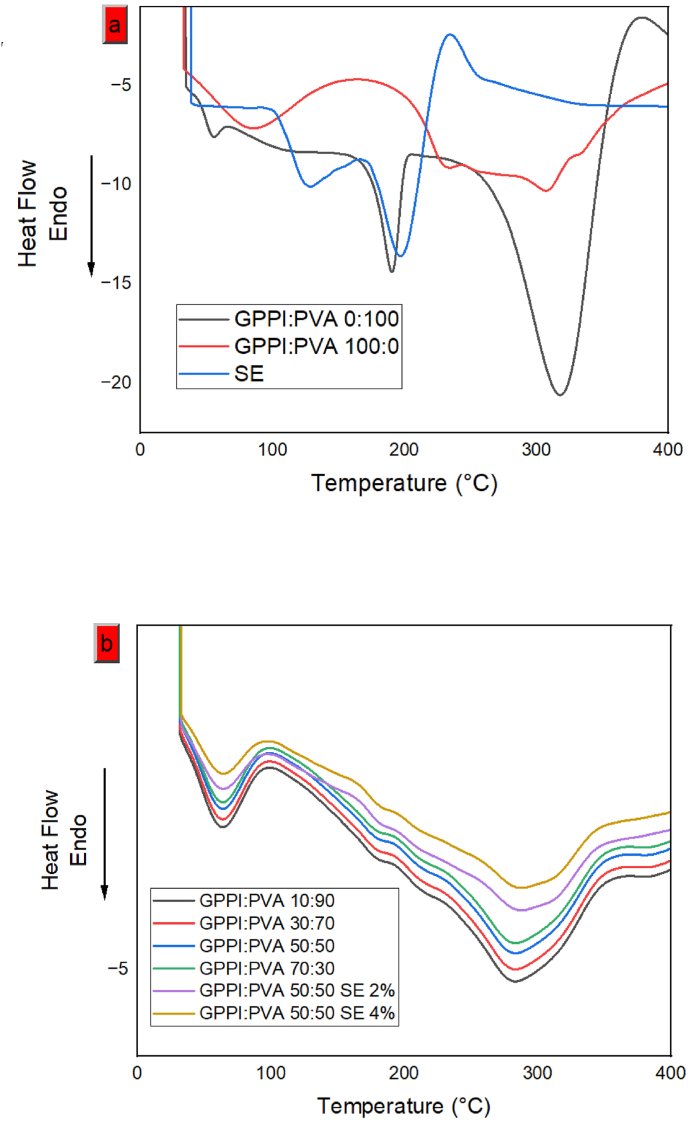
DSC curves of (a) PVA, GPPI, SE and (b) nanofibers with different ratios of GPPI:PVA, and GPPI:PVA nanofibers with different concentrations of SE. PVA: Polyvinyl alcohol, GPPI: grass pea protein isolate, and SE: sumac extract.

The thermal behavior and phase transition of a material can be obtained using DSC. The glass transition temperature (T_g_) and melting temperature (T_m_) are significant characteristics that can be derived from DSC data. The melting point is caused by the breaking of hydrogen bonds in the triple helix (Cetinkaya et al., 2024). The DSC curves of different samples are presented in Fig. 5. PVA exhibited 3 endothermic peaks, namely the glass transition at 57.82 ^°^C, melting at 193.59 ^°^C, and decomposition at 322.27 ^°^C (Shanesazzadeh et al., 2018; Moradinezhad et al., 2023).

The DSC curve of GPPI exhibited the denaturation peak at 87.97 ^°^C and melting peak at 232.90 ^°^C. Also, there was a wide area from 25 ^°^C to 143.49 ^°^C; this is related to the loss of water and the glass transition temperature Tg caused by the transition from a triple helix to a random coil. These findings are similar to the results reported by Ghorani et al. (2020), and Koosha et al. (2017), for gelatin nanofibers. In the hybrid nanofibers, by increasing the amount of GPPI, the melting point was enhanced, and the fibers became more heat-resistant. This is due to hydrogen bonding between the amino and carboxyl groups of GPPI and the hydroxyl group of PVA (Aguilar-Vázquez et al., 2018). SE raised the degradation temperature (from 280 to ∼293 ^°^C) by influencing the stability of the triple helix structure in the polymer through improved hydrogen bonding and hydrophobic interaction. The alteration in the thermal stability is attributed to the molecular interactions between GPPI:PVA and SE. SE was found to be in an amorphous state with good incorporation and no crystallization in the GPPI:PVA nanofibers, according to the thermograms (Cetinkaya et al., 2024). These findings were in accordance with the results of Pakseresht et al. (2023), who reported an increase in the thermal stability of the Cerish fructan-composite films by adding sumac extract. Also it has been reported that the addition of red grape anthocyanin to salep/cellulose aerogel (Mirmoeini et al., 2023) and black chickpea protein isolate nanofibers containing black chickpea peel anthocyanins (Amjadi et al., 2024) resulted in a rise in thermal degradation temperature. This phenomenon is due to the formation of new bonds between the anthocyanin and the film matrix.

### 3.6 Mechanical properties

The mechanical characteristics of nanofibers are presented in Table 2. Tensile strength (TS) is an indication of the mechanical stability of a material against external stress (Dey et al., 2020). While elongation at break (EAB) measures the tolerance of the material against bending or shaping before the material breaks. The highest TS (3.331 MPa) and EAB (37.558%) were measured in the PVA. While the nanofibers with high ratios of GPPI had low TS and EAB. The TS and EAB of GPPI:PVA with ratio 70:30 nanofibers were 0.025 and 4.541%, respectively, indicating very weak mechanical properties. These values were increased to 0.257 and 17.366 in GPPI:PVA with a ratio 50:50 nanofibers. Therefore, the increase in GPPI ratio decreased the mechanical properties of nanofibers. Aguilar-Vázquez et al. (2018), expressed that the presence of pea protein did not enhance the mechanical characteristics of pea protein/pullulan nanofibers but instead weakened the strength of pullulan nanofibers. But in another study Gutschmidt et al. (2021), showed that adding soy protein isolate to the PVA polymer mixture enhanced the tensile strength, modulus, and failure strain of the mats. Similar results were reported by Yao et al. (2022), for the electrospinning of peanut protein isolate/poly-L-lactic acid. Their study demonstrated that the tensile strength of the peanut protein isolate/poly-L-lactic acid composite nanofibers decreased as the peanut protein isolate content increased. The incorporation of SE into the composite nanofiber mats decreased their thickness and TS while increasing their EAB. These findings suggest that the inclusion of SE resulted in a reduction in mechanical strength and an increase in the flexibility of fiber mats. The reduction of TS and the increase in EAB could be explained by the inhibitory effect of SE on the interaction between GPPI:PVA and the potent plasticization of the phenolic components of SE (Pourjavaher et al., 2017). Heydarian and Shavisi (2023) observed a similar trend when blackberry anthocyanin was added to gelatin-xanthan gum nanofibers. While another study demonstrated that enclosing blueberry anthocyanin in wheat gluten protein films led to an increasing and then decreasing trend in TS (Hu et al., 2024).

**Table 2:** The effect of different ratios of GPPI:PVA and SE on thickness and mechanical properties of the nanofibers.

Samples	Thickness (mm)	Tensile strength (MPa)	Elongation (%)
GPPI:PVA 0:100	0.247 ± 0.008 ^a^	3.331 ± 0.073 ^a^	37.858 ± 2.880 ^a^
GPPI:PVA 10:90	0.247 ± 0.007 ^a^	3.110 ± 0.086 ^b^	36.016 ± 3.864 ^a^
GPPI:PVA 30:70	0.203 ± 0.005 ^b^	2.208 ± 0.057 ^c^	29.000 ± 2.166 ^b^
GPPI:PVA 50:50	0.069 ± 0.006 ^c^	0.257 ± 0.039 ^d^	17.366 ± 1.001 ^c^
GPPI:PVA 70:30	0.023 ± 0.003 ^e^	0.025 ± 0.011 ^e^	4.541 ± 0.524 ^d^
GPPI:PVA 50:50 SE 2%	0.049 ± 0.008 ^d^	0.047 ± 0.009 ^e^	27.400 ± 2.410^b^
GPPI:PVA 50:50 SE 4%	0.040 ± 0.001^d^	0.051 ± 0.001 ^e^	26.640 ± 1.750 ^b^

PVA: Polyvinyl alcohol, GPPI: grass pea protein isolate, and SE: sumac extract. Different letters on the same column exhibited significant differences (*P* < 0.05).

### 3.7 Antioxidant activity of fibers

**Fig. 6:**
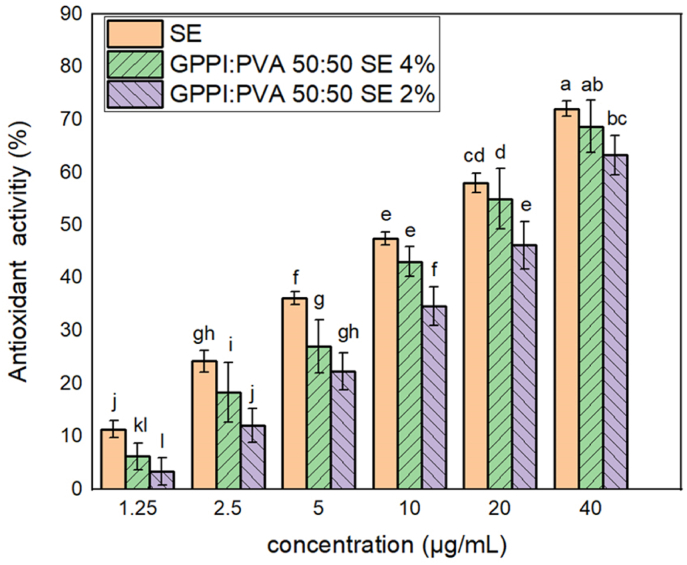
Antioxidant activity of SE and GPPI:PVA nanofibers with different concentrations of SE. PVA: Polyvinyl alcohol, GPPI: grass pea protein isolate, and SE: sumac extract.

Fig. 6 exhibits the antioxidant activity of SE and GPPI:PVA nanofibers with 2% and 4% SE. The antioxidant activity of both SE and GPPI:PVA nanofibers with SE were observed to increase as their concentration rose from 1.25 to 40 μg/mL. This enhanced antioxidant activity can be attributed to the presence of surface hydroxyl groups in sumac. The movement of hydrogen atoms from the hydroxyl groups in sumac to DPPH radicals caused the formation of deactivated DPPH-H compounds. The free radicals then regain stability by bonding with one another, thereby inhibiting the deactivation of DPPH free radicals (Yao et al., 2020). Pakseresht et al. (2023), and Cikrikci Erunsal et al. (Cikrikci Erunsal et al., 2023), demonstrated that sumac exhibited antioxidant activity against DPPH free radicals. They found that higher concentrations of sumac in films positively influenced the reducing ability and free radical-scavenging activity.

### 3.8 Antimicrobial activity

The antibacterial activity of the electrospun nanofibers was assessed by measuring the diameter of the inhibition zone; the results are shown in Table 3. It was observed that the sumac extract exhibited antimicrobial properties against *E. coli* and *S. aureus* with inhibition zones of 12 and 14 mm, but the control nanofiber (GPPI:PVA) did not display any inhibitory effects. Despite displaying reasonable antioxidant activity, GPPI:PVA with ratio 50:50 SE 2% failed to hinder the growth of any of these microorganisms. Nevertheless, regardless of the concentration of sumac in composite nanofibers, no inhibitory effect was detected against *E. coli*. However, the GPPI:PVA 50:50 SE 4% exhibited antimicrobial properties against *S. aureus,* and the inhibition zone was 10 mm. Generally, Gram-positive bacteria have a single glycoprotein-based membrane, while Gram-negative bacteria have a double membrane based on lipopolysaccharides, providing additional barrier properties against plant extracts. The lipid phase of the membrane may reduce the solubility of antimicrobial compounds, leading to resistance to inhibition effects (Aydogdu Emir, 2023).

**Table 3:** The antibacterial activity of SE, and GPPI:PVA nanofibers with a ratio of 50:50 without and with 2%, and 4% SE.

Sample		Inhibition zone (mm)	
		*Escherichia coli*	*Staphylococcus aureus*
SE		12	14
GPPI:PVA 50:50		ND	ND
GPPI:PVA 50:50 SE 2%		ND	ND
GPPI:PVA 50:50 SE 4%		ND	10

PVA: Polyvinyl alcohol, GPPI: grass pea protein isolate, and SE: sumac extract.

Previous studies demonstrated that SE can hinder the growth of numerous bacteria, with a higher efficacy observed against Gram-positive bacteria compared to Gram-negative bacteria. The research conducted by Emir et al. (2023), corroborated our findings on the antimicrobial properties of sumac and confirmed the concentration-dependent antibacterial effects of SE against various bacteria. Additionally, Mahdavi et al. (2018), reported that the ethanolic extract of Iranian sumac shows the highest antimicrobial effects against *S. aureus*, while *E. coli* was the most resistant.

## 4 Conclusion

In this study, the active nanofiber mats composed of GPPI and PVA as nanofiber matrix and SE as a bioactive agent were successfully fabricated by electrospinning technique. As the GPPI content increased, the diameter of nanofibers decreased due to the increase in electrical conductivity and decrease in viscosity. Nevertheless, the blends containing more than 70% GPPI resulted in beaded electrospun fibers. By incorporating the SE, the fibers became thinner, so that the nanofibers with 4% SE exhibited the smallest average diameter (105.8). FTIR and XRD results confirmed the successful loading of SE into the GPPI:PVA nanofiber. The electrospun GPPI:PVA:SE nanofibers exhibited better thermal stability than those without SE. The GPPI:PVA:SE nanofibers showed antioxidant and antimicrobial activity. It is recommended that future research focus on the physicochemical and mechanical characteristics of protein-based nanofibers and their applications. Therefore, the GPPI:PVA nanofiber mats with a ratio of 50:50 and 4% SE may serve as an effective active nanofiber to enhance the shelf life of food items.

## CRediT authorship contribution statement

**Marzieh Rezaei:** Conceptualization, Methodology, Software, Formal analysis, Investigation, Data curation, Writing – original draft. **Nasser Sedaghat:** Conceptualization, Validation, Resources, Writing – review & editing, Supervision, Project administration, Funding acquisition. **Sara Hedayati:** Conceptualization, Methodology, Validation, Resources, Writing – review & editing, Supervision, Project administration. **Mohammad-Taghi Golmakani:** Validation, Resources, Writing – review & editing, Funding acquisition.

## Funding

This work was supported by Ferdowsi University of Mashhad, Mashhad, Iran, grant number 3/59652.

## Declaration of competing interest

The authors declare that they have no known competing financial interests or personal relationships that could have appeared to influence the work reported in this paper.

## Data Availability

Data will be made available on request.
